# Cost analysis of the development and implementation of a spatial decision support system for malaria elimination in Solomon Islands

**DOI:** 10.1186/1475-2875-13-325

**Published:** 2014-08-18

**Authors:** Luke Marston, Gerard C Kelly, Erick Hale, Archie CA Clements, Andrew Hodge, Eliana Jimenez-Soto

**Affiliations:** Pacific Malaria Initiative Support Centre, National Vector Borne Disease Control Programme, PO Box 2119, Honiara, Solomon Islands; School of Population Health, The University of Queensland, Public Health Building, Herston Road, Herston, Brisbane, QLD 4006 Australia; Ministry of Health and Medical Services, Vector Borne Diseases Control Programme, Honiara, Solomon Islands; Research School of Population Health, College of Medicine, Biology and Environment, The Australian National University, Canberra, ACT Australia

**Keywords:** Malaria elimination, Cost analyses, Surveillance, Geographic information systems, Spatial decision support systems

## Abstract

**Background:**

The goal of malaria elimination faces numerous challenges. New tools are required to support the scale up of interventions and improve national malaria programme capacity to conduct detailed surveillance. This study investigates the cost factors influencing the development and implementation of a spatial decision support system (SDSS) for malaria elimination in the two elimination provinces of Isabel and Temotu, Solomon Islands.

**Method:**

Financial and economic costs to develop and implement a SDSS were estimated using the Solomon Islands programme’s financial records. Using an ingredients approach, verified by stakeholders and operational reports, total costs for each province were quantified. A budget impact sensitivity analysis was conducted to investigate the influence of variations in standard budgetary components on the costs and to identify potential cost savings.

**Results:**

A total investment of US$ 96,046 (2012 constant dollars) was required to develop and implement the SDSS in two provinces (Temotu Province US$ 49,806 and Isabel Province US$ 46,240). The single largest expense category was for computerized equipment totalling approximately US$ 30,085. Geographical reconnaissance was the most expensive phase of development and implementation, accounting for approximately 62% of total costs. Sensitivity analysis identified different cost factors between the provinces. Reduced equipment costs would deliver a budget saving of approximately 10% in Isabel Province. Combined travel costs represented the greatest influence on the total budget in the more remote Temotu Province.

**Conclusion:**

This study provides the first cost analysis of an operational surveillance tool used specifically for malaria elimination in the South-West Pacific. It is demonstrated that the costs of such a decision support system are driven by specialized equipment and travel expenses. Such factors should be closely scrutinized in future programme budgets to ensure maximum efficiencies are gained and available resources are allocated effectively.

**Electronic supplementary material:**

The online version of this article (doi:10.1186/1475-2875-13-325) contains supplementary material, which is available to authorized users.

## Background

Malaria in the Pacific region has been an essential component of the global health agenda since the 1950s. More recently, considerable renewed interest has developed towards malaria elimination [1]. Global investment is now greater than ever at approximately US$ 2.5 billion in 2012 [2], with recent estimates of funding required to meet the Global Malaria Action Plan objectives at approximately US$ 4-6 billion annually [3–7]. The efficient utilization of these resources is paramount, and further evidence on the costs and benefits of malaria elimination, and the tools to achieve the optimal allocation of resources are required [4].

The management and control of this global disease has seen numerous significant achievements matched equally with disappointments [8–10]. Regrettably, history has shown the potential fragility of hard-fought gains [11]. The Solomon Islands is a case in point. Following the success of ‘near’ eradication during the 1970s, the situation deteriorated to the point that the country held the disreputable title of the highest malaria incidence in the Asia-Pacific Region in the 1990s [9, 12–17]. A regional initiative was launched by the Australian Government in 2008 to address this disease burden, along with funding and support from other donors and stakeholders, including the Global Fund To Fight AIDS, Tuberculosis and Malaria, Solomon Islands Government, World Health Organization (WHO) and Japanese International Cooperation Agency [18, 19]. It has been generally agreed that the fight against malaria in the Pacific region must be renewed with energy, armed with the latest tools and strategies aimed at scaling up national malaria programmes to a ‘pre-elimination’ stage by 2014 [6, 20, 21].

Since 2008, the Solomon Islands’ National Vector Borne Disease Control Programme (NVBDCP) has embarked on a programme of aggressive malaria control. With a markedly low incidence of malaria transmission in the provinces of Temotu and Isabel, pilot malaria ‘elimination’ programmes commenced in these locations [18, 20, 22]. In these provinces, malaria transmission has occurred in foci of geographically centred events, and the programme is moving towards intensive surveillance, and detailed case investigation and screening of asymptomatic populations in order to clear any parasitic reservoir in the population [15, 22–25]. This process of surveillance is coupled with scaled-up frontline interventions including indoor residual spraying, long-lasting insecticide-treated net distribution and community awareness campaigns [22]. In line with the WHO’s recommendations [21], strategic objectives were developed to best utilize geo-referenced data to support the programme’s capacity to effectively manage scaled-up interventions at a level of detail that is required for malaria elimination; as well as implement high-resolution surveillance and guide the targeting, planning and effective implementation of response interventions to limit the further transmission of malaria. This has led to the development of a geographical information system (GIS) based spatial decision support system (SDSS) [18, 26]. This system has been outlined in detail elsewhere, and generally has been found to be a user-friendly approach to support surveillance, monitoring and evaluation [26, 27].

A substantial literature exists on the cost dynamics of malaria programmes [28], particularly on the costs and cost-effectiveness of traditional interventions [29]. The vast majority of recent studies analyse interventions throughout sub-Saharan Africa, with only limited evidence from the Asia-Pacific region [12, 30–33]. Little cost data are available on recently developed innovations, such as the use of ‘surveillance’ as a tool in eliminating malaria. Whilst the literature strongly supports the use of geo-spatial tools to support scaled-up elimination campaigns [34–37], virtually no evidence exists on the costs associated with geographical information based systems, such as the SDSS, particularly in the context of resource-poor environments. Consequently, only a weak evidence base exists to inform ongoing decisions required in the scale up of elimination activities.

The present analysis examines the costs of the development and implementation of a SDSS for malaria elimination in two provinces of the Solomon Islands. Whilst this work does not provide either cost effectiveness or cost-benefit analyses, it does provide a clear picture of the investment required in developing this new tool for malaria elimination and provides the basis for future cost analyses. The analysis also assesses the degree to which variations in the design of the intervention influence programme costs. Other countries in the region with national policies of malaria elimination may potentially utilize this cost information in the planning and resource allocation process when developing their strategies and making operational decisions for malaria control and elimination.

## Methods

### Description of the SDSS in the Solomon Islands

The Solomon Islands’ NVBDCP established the SDSS in mid-late 2008, with the goal of improving planning, implementation, and monitoring and evaluation of malaria interventions, as well as managing the more detailed malaria case data (to the household level) for malaria elimination. The programme’s design, technical specifications and applications are detailed elsewhere [20, 22, 26, 27]. In brief, a SDSS is an integrated database management system that provides computerized support for decision-making where there is a geographic or spatial component available. This computer-based information system utilizes routinely available data collected as part of standard surveillance, monitoring and evaluation activities. The SDSS in the Solomon Islands utilizes the existing, paper-based data management framework. However, it now requires the consulting clinician to report an additional data field, the patient’s household number. This information is then entered into a Microsoft Access® database. With this case data now linked to a geo-referenced household register, the SDSS utilizes a customized version of the GIS software programme MapInfo Professional® to identify malaria cases in a geographic context, where case data is electronically superimposed on topographical maps of each province.

Following an intensive planning process, the development and implementation of the SDSS involved training of local staff, the purchase of required equipment and materials, and the collection of geo-reference data at the household level (referred to as geographical reconnaissance). Concurrently, the development of a central-level database was undertaken, sharing common units and data structures where possible, while allowing the information to remain applicable to locally sensitive needs. Updates and modifications were undertaken to ensure maximum applicability of the SDSS to local needs [26, 27, 38].

The SDSS was implemented in two different geographical locations – Isabel Province and Temotu Province (see Additional file 1: Figure S1). This required the development of two separate information systems, which utilized the same software, systems and basic structure to produce locally specific information. Geographical reconnaissance in Temotu Province took place from September-November 2008 and from March-May 2010 in Isabel Province. The reconnaissance required two small teams of two to three people in Temotu Province. Due to the logistical difficulties and remoteness of the populated outer islands, the teams were part of a broader baseline malaria survey team, which hired a live-aboard research vessel to conduct a parasite prevalence and entomological distribution study [39]. Due to the limited availability of the research vessel, the team was required to finalize the reconnaissance activities without the vessel in February 2009 on the main island of Santa Cruz. For Isabel Province, a ‘mapping officer’ was attached to four separate indoor residual spraying teams to conduct the geo-referenced data collection. Once the reconnaissance was completed and the local databases established, routine passive case detection data from provincial health facilities was entered into the database by province-based malaria programme staff. The SDSS can produce numerous outputs, including a geographical summary of the distribution of malaria, trends in malaria transmission as well as descriptive maps of the distribution and coverage of related interventions (see Additional file 1: Figure S2-S4).

Table 1 presents important operational parameters defined during the roll out of the SDSS. Whilst the provinces were comparable in terms of population size and number of households, key differences were evident in their geography/topography, which impacted upon implementation of the SDSS. Note that the Temotu Province is both the most easterly province of Solomon Islands and the most remote, with five islands groups stretching over a sea area of approximately 130,000 km^2^.

**Table 1:** **Key operational parameters for SDSS development and implementation in Temotu Province and Isabel Province**

Operational parameter	Temotu Province	Isabel Province
Total number of households mapped	5,221	6,368
Total number of structures mapped	11,874	12,787
Population (2011 SI Census)	21,552	30,176
Land Area (km^2^)	836	4,120
Number of days to complete GR	37	45
Households mapped per day	141.11	141.51
Teams conducting GR	2 (2-3 persons)	4 (1 person)
Population Density - land (pop'n per km^2^)	25.78	7.32

*Notes:* Data were sourced from published articles [26, 27] and internal activity reports in the NVBDCP.

### Costing

The total economic cost of the development of the SDSS is based on a five-year implementation period. This time span was indicative of the period from the decision to implement an SDSS (August 2008) through to full operation (August 2013); from which time the SDSS was assumed to be fully operational in its intended capacity. The analysis was undertaken from a programme management perspective, which identified direct investment or administrative costs from stakeholders to support the development and implementation of the SDSS. These were considered additional costs outside of the routine expenses incurred by the NVBDCP for programme monitoring and reporting utilising the existing information system. The comparison (null) for this analysis was no SDSS, with the NVBDCP utilising the previously existing system. In an approach similar to other cost analyses of malaria interventions [40–42], an ingredients methodology was utilized [43]. Individual transactions were reviewed and inputs were confirmed through available receipts and verified by the implementing stakeholders in terms of the purpose of items purchased and/or activities conducted [44]. Opportunity costs were considered minimal as the SDSS is essentially a refinement of an existing system.

Detailed retrospective direct programme cost data were collected from financial records of the key budgetary stakeholders. This included accounting costs recorded in transaction listings from the Solomon Islands Ministry of Health and Medical Services (MHMS), and from financial records of the Pacific Malaria Initiative Support Centre (PacMISC). When financial costs were unavailable for specific SDSS-related activities, MHMS or PacMISC records were sourced for unit costs values and quantities for various inputs.

Costs were identified in either Australian Dollars (AUD) or Solomon Islands Dollars (SBD). These costs were converted to US Dollars using historical exchange rates [45], and were inflated/deflated to provide constant 2012 US Dollar amounts using the United States’ average annual consumer price index figures [46]. Capital costs were listed over the five-year period, and a 3% discount rate applied as per WHO recommendations [47]. In maintaining a simplified approach, no wastage factor was applied to such resources as computers, PDAs, fuel or other items. This was in part due to the fact that in the procurement of many items identified in the financial records a ‘wastage’ component was often considered in the original purchase. For example, buffer amounts of materials and equipment and/or fuel were purchased in the first instance due to the logistical constraints, isolation and limited access to readily available resources in the Solomon Islands.

In cases where costs were identified as having a mixed purpose (e.g. training for IRS and use of the SDSS for planning and reporting), a proportional allocation of each budgetary component was estimated based on the purpose and contribution specific to the SDSS. Proportional allocations were done through review by implementing stakeholders, and based on criteria including the main purpose or objective of the item or activity, the mix of staff and personnel involved in the activity, and the units and/or quantities of resources utilized specifically for the SDSS. Supporting documentation such as receipts, field reports and meeting/workshop minutes were used to support the proportional allocation to the overall cost for the SDSS development.

Total costs were classified under two separate categories. First, costs were categorized into standard budgetary resource components utilized by the Solomon Islands’ Government. Where expenses were identified through an ‘imprest’ payment, the imprest budget and/or acquittal were used to identify amounts under each budgetary code. Secondly, costs were also categorized into start-up, geographical reconnaissance, or ongoing management expenses of the SDSS (see Additional file 1: Table S1 for more details). This process was done through consensus with programme staff and key implementing stakeholders on review of the costs identified, and estimation of routine costs moving forward.

Cost data were tabulated in Microsoft Excel®, where summaries and cross-tabulation with cost categories and SDSS development phases provided a concise and detailed account of costs. A list of cost categories, unit costs and other relevant assumptions can be found in the supplementary information (see Additional file 1: Table S2). Because the SDSS did not have a direct link to the communities or individuals who access services and the malaria interventions, patient costs and/or consumer costs are not considered in this analysis.

### Sensitivity analyses

To obtain an understanding of the individual cost components and how variations in the input parameters may impact future budgetary allocations for the roll out of the SDSS in other malaria elimination sites, a univariate sensitivity analysis of cost components was performed. The analysis was conducted on cost data from each province, in line with previous cost analyses undertaken on malaria interventions [40, 42]. Each cost category’s unit costs were increased/decreased by 20% to test their influence on the total cost in each province. In line with the WHO’s Guide to Cost-effectiveness Analysis, discount rates were tested at 0% and 5% [47]. An alteration to the implementation model was also tested regarding the supervisory support visits, which at baseline included a site visit each year. This was tested with visits in only the first two years, assuming the integration of visits in years three, four and five within routine surveillance and evaluation activities. Results are displayed in a tornado diagram for each province. Ethical approval for access to financial data and analysis was obtained from the University of Queensland, School of Population Health Research Ethics Committee, and the Solomon Islands National Health Research and Ethics Committee.

## Results

The total cost for the five-year development and implementation of the SDSS was US$ 96,046 (2012 constant dollars), with US$ 49,806 for Temotu Province and US$ 46,240 for Isabel Province. The single largest cost component was for equipment to implement the SDSS (e.g. laptop computers, GIS-enabled PDAs and peripherals), accounting for over 30% of the total cost. Other significant cost categories included software licenses at approximately 14%, as well as expenses associated with travel (e.g. accommodation, travel fares and staff per diem), which combined totalled almost 40%. All costs by category and by province are detailed in Table 2.

**Table 2:** **Total financial and economic costs of development and implementation of SDSS for malaria elimination in Solomon Islands**

Resource	Temotu Province		Isabel Province		Total
	Total cost	%	Total cost	%	
Salaries	$1,484.10	(1.55)	$1,484.10	(1.55)	$2,968.21
Allowances	$8,391.23	(8.74)	$4,723.40	(4.92)	$13,114.63
Fuel	$4,800.08	(5.00)	$3,738.00	(3.89)	$8,538.09
Accommodation	$10,494.04	(10.93)	$2,767.99	(2.88)	$13,262.02
Other	$1,009.67	(1.05)	$1,658.46	(1.73)	$2,668.13
Boat/OBM Hire	$315.30	(0.33)	$650.81	(0.68)	$966.11
Equipment	$10,061.48	(10.48)	$20,024.20	(20.85)	$30,085.67
Software license	$5,046.04	(5.25)	$8,410.07	(8.76)	$13,456.11
Travel fares	$8,204.48	(8.54)	$2,783.03	(2.90)	$10,987.51
**Total**	**$49,806.43**		**$46,240.06**		**$96,046.49**

*Notes:* Dollar amounts are in 2012 constant $US.

The geographical reconnaissance phase of the SDSS development proved to be the most costly, with over 60% of total costs (i.e. US$ 59,770) associated with this activity. The ongoing management and support costs accounted for just under one-third of the overall costs over the five-year implementation period. Costs by phase of development and implementation are described below in Table 3. Ratios of costs for start-up, geographical reconnaissance, and ongoing management and support were comparable for both provinces, and consistent with aggregated figures.

**Table 3:** **Total costs by SDSS development and implementation phase**

Phase	Temotu Province		Isabel Province		Total
	Total cost	%	Total cost	%	
Site visit and staff orientation	$3,425.33	(3.6)	$2,800.63	(2.9)	$6,225.96
Geographical Reconnaissance	$31,621.83	(32.9)	$28,175.01	(29.3)	$59,796.83
Ongoing Management and Support	$14,759.27	(15.4)	$15,264.42	(15.9)	$30,023.69
**Total**	**$49,806.43**		**$46,240.06**		**$96,046.49**

*Notes:* Dollar amounts are in 2012 constant $US.

### Budget impact sensitivity analysis

**Figure 1:**
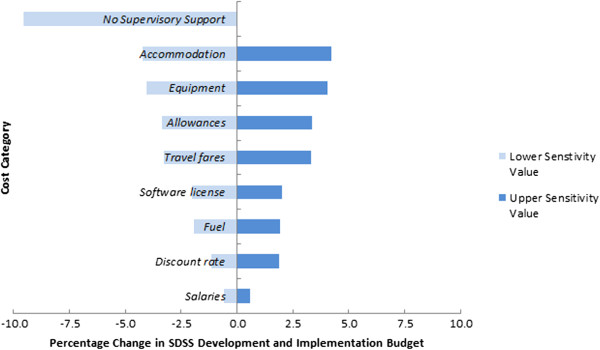
**Sensitivity analysis, variation in cost components, Temotu Province.**

**Figure 2:**
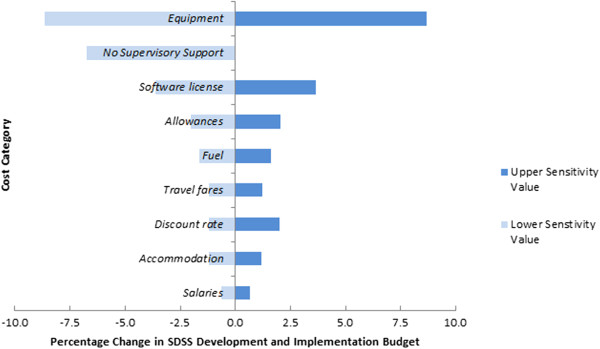
**Sensitivity analysis, variation in cost components, Isabel Province.**

A summary of cost categories, unit costs and values included in the sensitivity analysis are detailed in Table 4. Figures 1 and 2 display the results of the sensitivity analysis for each province graphically in a tornado diagram for overall budget impact.

**Table 4:** **Sensitivity analysis of costs of SDSS**

Parameter	Variation	Temotu		Isabel	
Baseline result:		$49,806.43		$46,240.06	
		Lower value	Upper value	Lower value	Upper value
Salaries	+/- 20%	$49,509.61	$50,103.25	$45,943.24	$46,536.88
Allowances	+/- 20%	$48,128.18	$51,484.68	$45,295.38	$47,184.74
Fuel	+/- 20%	$48,846.41	$50,766.45	$45,492.46	$46,987.66
Accommodation	+/- 20%	$47,707.62	$51,905.24	$45,686.46	$46,793.66
Equipment	+/- 20%	$47,794.14	$51,818.73	$42,235.22	$50,244.90
Software licenses	+/- 20%	$48,797.22	$50,815.64	$44,558.05	$47,922.07
Travel fares	+/- 20%	$48,165.53	$51,447.33	$45,683.45	$46,796.67
Discount rate	0%, 5%	$49,231.69	$50,753.57	$45,684.78	$47,158.03
Supervisory Support	No supervisory support visits conducted in years 3, 4 and 5	$45,036.34	$49,806.43	$43,124.30	$46,240.06

*Notes:* Dollar amounts are in 2012 constant $US.

Reducing the number of supervisory support visits had a major impact in both provinces. The change could potentially cater for a 9.6% and 6.7% budget saving in total SDSS costs in Temotu and Isabel, respectively. In Temotu Province, changes in equipment costs demonstrated significant overall budget impacts with 4% overall budget savings with reduced unit costs. In light of the challenging logistical setting in Temotu Province, and its isolation and remoteness from the capital (i.e. Honiara), travel costs had a contributable influence on the budget, with a combined impact of approximately 11%. Fuel costs, salaries and changed discount rate all presented a budget impact of less than 2%.

In Isabel Province, equipment costs were also found to be the most sensitive component, with approximately 9% savings potential achieved with reduced equipment costs. Software licenses and their annual renewal fees were found to have an almost 4% impact. Given the closer proximity of the Isabel Province to Honiara and the approach to undertake reconnaissance, variation in travel costs for programme staff had smaller impacts on the overall budget costs.

## Discussion

This study presents the first cost analysis of the implementation of a SDSS designed to facilitate the elimination of malaria in the Pacific region. The current analysis estimates that the total cost of developing and implementing the SDSS is US$ 96,046. The largest cost components were equipment and travel expenses. The sensitivity analysis highlighted that cost savings depend on the characteristics of the province. In more remote areas, the intervention would be more affordable if changes in travel costs were achieved, while reduced equipment costs would deliver considerable budget savings more generally.

Provincial-level costs are valuable information required for accurate programme planning within any context, but particularly in the Solomon Islands, where limited evidence of costs exists. This analysis demonstrates organizational structures and the subsequent allocation of equipment can have a considerable impact on the total budget required for malaria elimination activities. As the malaria elimination programme continues in the Solomon Islands and elsewhere, the roll out of any SDSS in other contexts would require careful consideration of the costs related to the computerized equipment, especially in larger provinces with a greater number of operational zones. Recent advances in the capacity and accessibility of technology, and the ubiquity of GIS-enabled portable devices has seen rapid changes in the market [27]. In fact, considering market changes, the 20% adjustment in unit prices assumed in the sensitivity analysis was conservative. Prices sourced in October 2013 from Australian retailers [48, 49] for computerized equipment with the capacity to perform functions required for the SDSS (e.g. lower range laptop US$ 450, tablet with GIS capability US$ 350 and portable printer USD $150) could deliver a reduction in the unit cost for equipment as a package to the amount of 50%. Moreover, the increased availability of open-source GIS software (e.g. Quantum GIS) could further reduce software costs, although such costs were not substantial. Nonetheless, the potential for further savings in the SDSS total cost is clearly evident.

It is now widely accepted that geo-referenced case data are integral to inform malaria elimination [21, 37]. An important step in the development of any high-resolution SDSS for malaria elimination entails access to geo-referenced household data, which has become more accessible due to high-quality data collection systems with the advancement of GIS-based technologies and their role in programme operations, surveys and census as well as the allocation of additional resources to do this kind of work [26]. The associated costs to develop these information systems, and in particular the investment required at each phase was previously not well known. Results indicate the geographical reconnaissance phase for the SDSS development in Solomon Islands accounted for approximately 62% of the total cost (US$ 31,622 Temotu Province, US$ 28,175 Isabel Province). Hence, had suitable geo-referenced household data been previously available, a considerable cost saving could have been made. Moreover, this study is based on an ‘blanket approach’ to household mapping at the provincial level, however, more recent studies indicate geographical reconnaissance may be focused at a sub-provincial level (i.e. around transmission foci) [34, 50, 51], which may reduce the overall costs for future provinces considering malaria elimination in the future.

Ongoing management and support costs accounted for approximately 32% of the total cost. In countries heavily dependent on international aid for service delivery, such as the Solomon Islands, this poses policy questions regarding the financial feasibility of malaria elimination for both the international donor and the recipient. This applies not only for the SDSS, but potentially other information management tools [52].

Whilst the cost analysis attempts to capture all costs relevant to the SDSS development and implementation, some irregular costs were part of the analysis. For example, a research vessel was hired as part of a broader baseline malaria survey in the outer islands of the Temotu Province and used by the SDSS mapping teams. The mapping teams were accommodated on board the research vessel, providing a significant cost saving under the accommodation line item. However, when costs were calculated based on an implementation model with standardized unit costs for accommodation, the cost of this proved to have a significant impact on the total budget (with a 20% reduction in the unit costs translating into a 4.2% budgetary saving). This becomes evident when comparing accommodation costs between Temotu and Isabel Province in the total cost summary.

The applicability of the costs for geographical reconnaissance and the SDSS in other jurisdictions should be interpreted with some caution because geographical and topographical variations in provinces as well as infrastructure – including the availability of electricity, access to boats and vehicles, ports and airstrips – could significantly influence the total investment required. Moreover, the different approaches undertaken to conduct the reconnaissance in each of the two provinces presented difficulties for the costing analysis. Costs for certain categories may have been shared, or in contrast, overly allocated to the SDSS budget. For example, in Isabel Province, where the mapping officers worked closely with spraying teams, the fuel costs may have been under- or over-estimated because they were shared between the two activities. Whilst attempts were made to correctly apportion the costs where information exists, uncertainty inevitably exists. More generally, the availability and reliability of cost data in Solomon Islands also represents a limitation of this study. Inadequate financial infrastructure and limited financial management capacity may have introduced bias to the cost estimates. They are, nonetheless, the best estimates available. Efforts were made to clean the cost data. Within-country experience over a number of years provided the lead author an opportunity to gain an in-depth understanding of the operational requirements and familiarity with the financial systems, potentially improving the quality of the data.

The SDSS is a new tool which has required investment in capacity building of local staff. This study does not include expenses associated with technical assistance, which is a limitation of the present analysis. This was due to the complexities of the design, delivery and support mechanisms for the development and implementation of the SDSS. Technical assistance expenses varied greatly between the provinces, with a greater focus on technical assistance support during the initial implementation of the SDSS. As systems were developed and local staff trained, an inherent capacity was built within the programme, with the need for international technical assistance (especially on-site) reducing significantly. This was evident on review of cost data, which indicated that stakeholder expenses associated with technical assistance in Isabel Province in 2010 were minimal. This in part may be due to the awareness and general increase in capacity of local staff following the experience in Temotu Province. Future cost analyses could investigate the role of capacity building and technical assistance in the development and implementation of the SDSS. For instance, the increased accessibility and reach of IT systems and equipment will no doubt improve the base capacity and operating efficiency to develop and implement a province-specific SDSS. On the other hand, as information systems are developed, IT requirements will increase (e.g. systems for data back up and networks to transfer of data from region to province and province to national levels). It is debatable whether this ‘ingredient’ contributes directly to the SDSS or whether it is a routine operational expense. For the purposes of this investigation, the latter has been assumed, and thus, IT systems were not included in the analysis. Future analysis of information systems should attempt to quantify the more complex costs associated with the SDSS development and implementation, including technical assistance.

## Conclusion

Long-term commitment to malaria elimination is required from donors, recipients and the international community [53, 54]. The high cost of health service delivery is a common characteristic in small Pacific nations [55, 56]. With per capita aid spending on malaria in the Solomon Islands one of the highest in the world [57], greater scrutiny of the most efficient tools to eliminate malaria is necessary [54]. SDSS are multifaceted tools with the ability to improve malaria surveillance, support operational planning and monitoring, and to more efficiently guide the interventions required for malaria elimination. This study presents the total costs and the key cost driving factors for the development and implementation of the SDSS in the first two provinces to embark on malaria elimination in the Solomon Islands. It forms the basis for further cost-effectiveness and cost-benefit analysis, which should focus on evaluating the operating efficiencies gained from the SDSS, and improvements in productivity. Further research on health care costs will contribute significantly to the evidence base, which is currently lacking for malaria interventions in the South-West Pacific.

## Electronic supplementary material

Additional file 1: Figure S1: Solomon Islands, Isabel Province, Temotu Province. **Figure S2**: Malaria cases, Santa Cruz Island, Temotu Province, 4^th^ Quarter 2011. **Figure S3**: Distribution of all confirmed malaria cases, Isabel Province, 2011. **Figure S4**: IRS coverage map vs. Households, Santa Cruz Island, Temotu Province, 2010. **Table S1**: SDSS Development Phases. **Table S2**: Cost details, unit costs, and assumptions for SDSS. (DOCX 538 KB)
