# Supplementary material for: Cost analysis of the development and implementation of a spatial decision support system for malaria elimination in Solomon Islands
Source: Malar J. 2014 Aug 18;13:325. doi: 10.1186/1475-2875-13-325 (PMC4148529; doi:10.1186/1475-2875-13-325)
Supplement: Supplementary file 1 — Additional file 1: Figure S1: Solomon Islands, Isabel Province, Temotu Province. Figure S2: Malaria cases, Santa Cruz Island, Temotu Province, 4th Quarter 2011. Figure S3: Distribution of all confirmed malaria cases, Isabel Province, 2011. Figure S4: IRS coverage map vs. Households, Santa Cruz Island, Temotu Province, 2010. Table S1: SDSS Development Phases. Table S2: Cost details, unit costs, and assumptions for SDSS. (DOCX 538 KB) [file 12936_2014_3359_MOESM1_ESM.docx]

**Additional file**

**Figure S1: Solomon Islands, Isabel Province, Temotu Province**


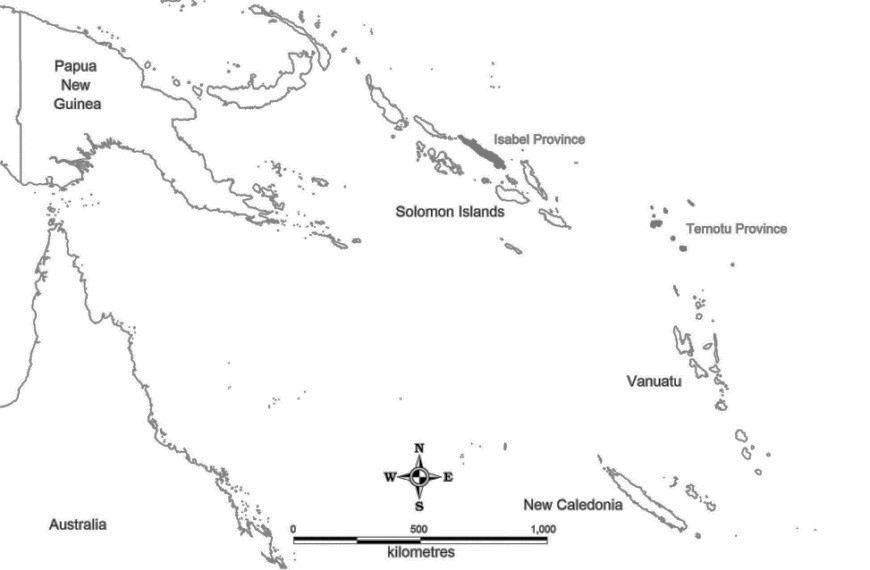


*Source:* NVBDCP Monitoring and Evaluation Unit, Solomon Islands Ministry of Health and Medical Services

**Figure S2: Fourth quarter 2011 malaria cases, Santa Cruz Island, Temotu Province**


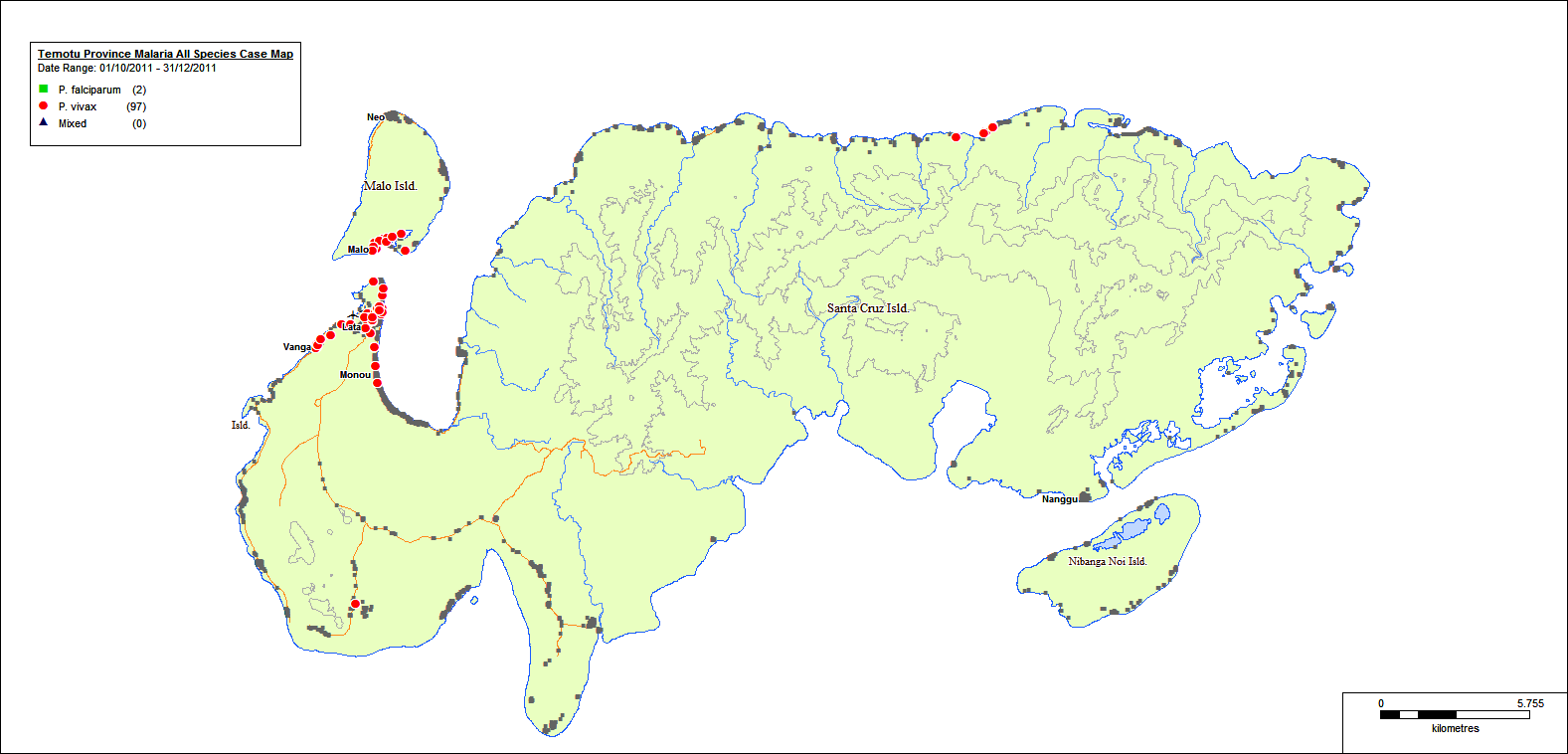
*Source:* NVBDCP Monitoring and Evaluation Unit, Solomon Islands Ministry of Health and Medical Services

**Figure S3: Distribution of all confirmed malaria cases, Isabel Province 2011**


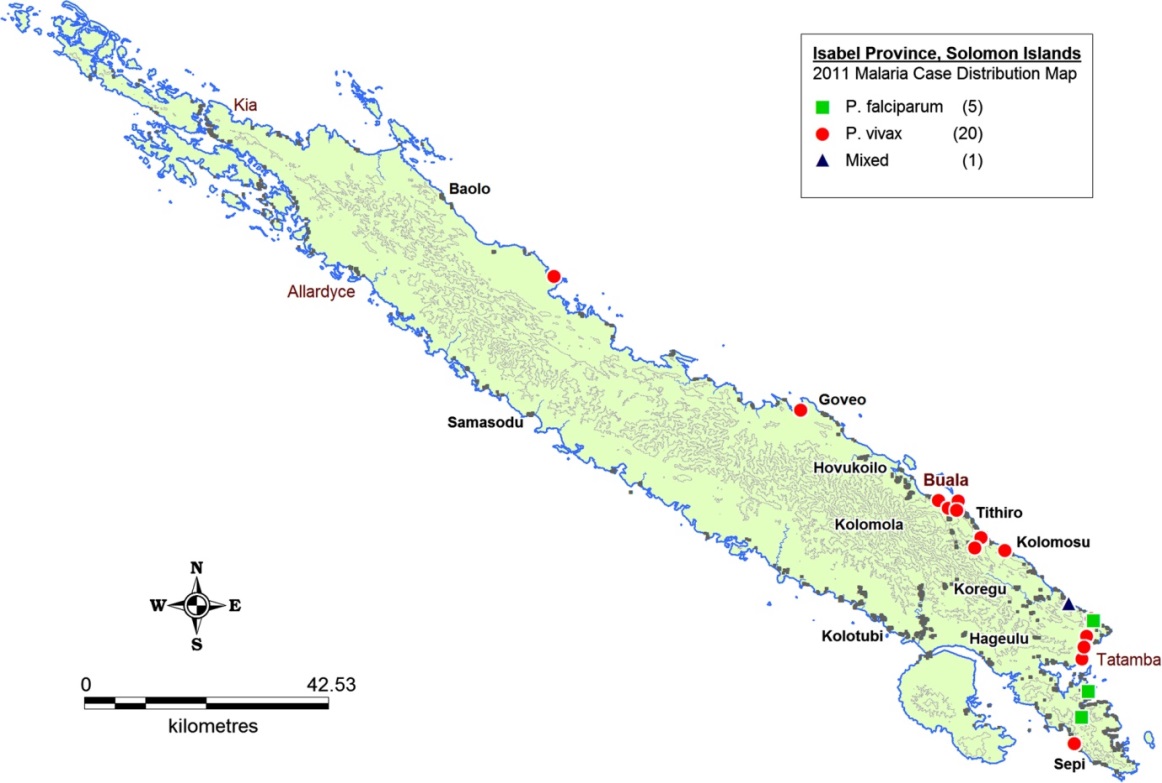


*Source:* NVBDCP Monitoring and Evaluation Unit, Solomon Islands Ministry of Health and Medical Services, 2013


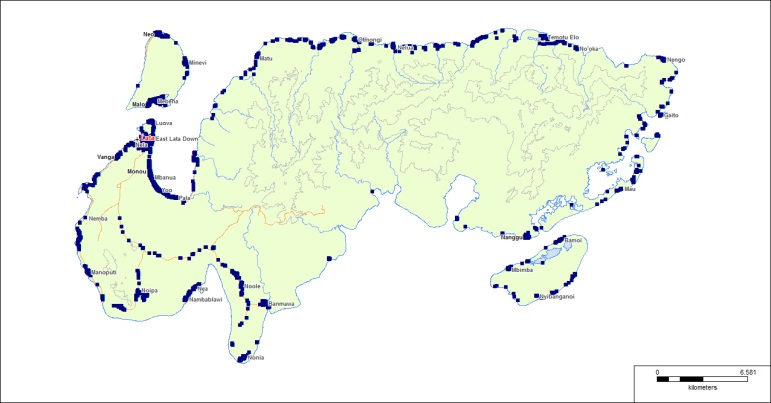

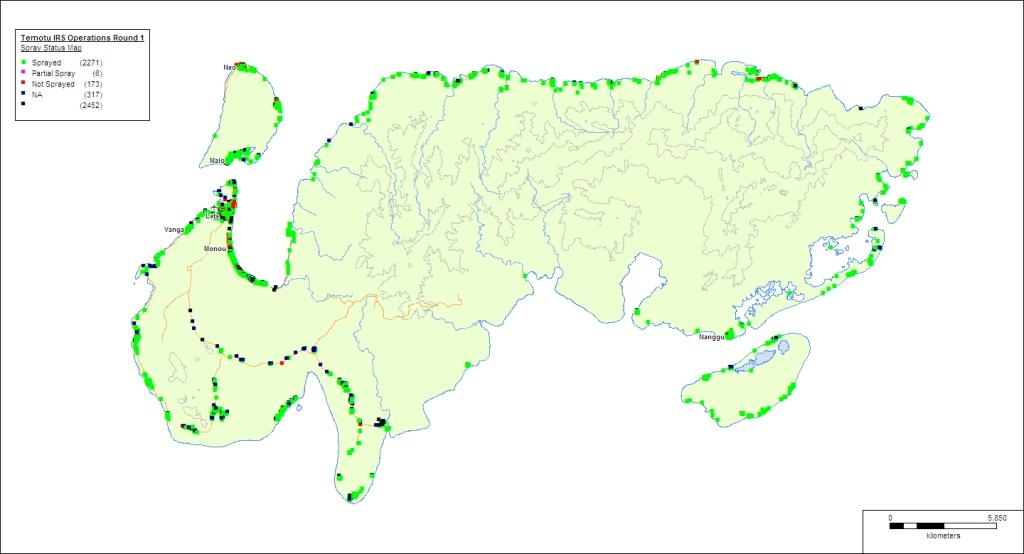
**Figure S4: IRS coverage map vs. Households, Santa Cruz Island, Temotu Province, 2010**

*Source:* NVBDCP Monitoring and Evaluation Unit, Solomon Islands Ministry of Health and Medical Services

**Table S1:** **SDSS Development Phases**

| **Phase** | **Description** |
| --- | --- |
| 1. Start up | Activities and items involved in the planning and preliminary events prior to the geographical reconnaissance and household enumeration exercise. For example:   - Reorientation of malaria information system - Objectives, needs, key features identified, 1 week situational assessment undertaken on-site - Initial design and concept development, macro-architecture of information system for malaria elimination - Logistical planning, identification of resources |
| 2. Geographical Reconnaissance | The purchase of equipment, obtaining software licenses, and training for geographical reconnaissance teams prior to undertaking the household mapping exercise. For example:   - Purchase/procurement of equipment and necessary for GR and for modified information system - Training of GR staff on data collection techniques, 1 week training on site - Household enumeration - Questionnaires - Logistics support - Household register developed - Database structured and populated - Back up and data management protocols developed |
| 3. Ongoing Development | Ongoing support and management costs were identified as transactions or expenses incurred specifically for maintenance, management and refinement of any component of the SDSS. For example:   - Supervisory support visit, 1 week - Recurrent procurement of software - Trouble shooting - Salaries and wages supporting management of SDSS with routine SM&E - Application of SDSS for spatial and temporal monitoring of malaria cases - SDSS used for detailed planning if malaria interventions and appropriate, efficient resource allocation |

*Notes:* SDSS development was categorised into the three distinct phases representing key activities completed in the process of developing a functional SDSS for malaria elimination

**Table S2: Cost details, unit costs, and assumptions for SDSS**

| **Units** | **Unit price estimates relevant to SDSS development and implementation cost analysis** |
| --- | --- |
| Fuel | Unit prices vary within Solomon Islands relative to the logistical expenses incurred in delivering fuel to a particular location, transport and infrastructure services available at the place of purchase. On review of financial records obtained between 2008-2011, fuel prices were conservatively estimated at 2008 SBD $20.00 per litre based on available receipts and budgets over this period. This 2008 value has been converted to a US dollar amount, with the USD amount inflated each year in line with US CPI inflationary figures. This yearly inflated USD amount has been used as the unit cost for fuel each year over the period of SDSS development and implementation. The table below indicates the yearly USD unit cost for fuel in Solomon Islands. It has been converted back to a SBD amount for reference:   \| **Year** \| **SBD cost per litre** \| **USD unit cost** \| \| --- \| --- \| --- \| \| 2008 \| $20.00 \| $2.74 \| \| 2009 \| $20.78 \| $2.73 \| \| 2010 \| $21.77 \| $2.77 \| \| 2011 \| $21.49 \| $2.86 \| \| 2012 \| $20.70 \| $2.92 \| |
| Salaries | The Solomon Islands Public Service Salary Structure indicates annual remuneration for Solomon Islands Government Employees. For the development and implementation of the SDSS, a redeployment of human resources was required by the NVBDCP Monitoring and Evaluation Manager. Following discussion and analysis of resources utilised for the SDSS, it was agreed that the NVBDCP Monitoring and Evaluation Manager was required to allocate one half-day per week (or 0.1 full time equivalent) on maintaining the geo-referenced database and supporting provincial teams to utilise the information obtained throughout the start-up and implementation period. The NVBDCP Monitoring and Evaluation Manager salary structure (sourced from the Solomon Islands Public Service Salary Structure), and its subsequent unit price is detailed below for the period 2008-2012.   \| **Year** \| **Annual salary (SBD)** \| **0.1 FTE Equiv (SBD unit cost)** \| **USD unit cost** \| \| --- \| --- \| --- \| --- \| \| 2008 \| $41,003.48 \| $4,100.35 \| $561.61 \| \| 2009 \| $42,848.63 \| $4,284.86 \| $562.82 \| \| 2010 \| $46,062.28 \| $4,606.23 \| $587.09 \| \| 2011 \| $47,904.77 \| $4,790.48 \| $637.91 \| \| 2012 \| $48,324.28 \| $4,832.43 \| $682.03 \|   As one manager covered both provinces (Temotu and Isabel) under his duties, it was assumed that each province maintained an equal share of resources dedicated from the M&E manager. |
| Allowances | Solomon Islands Government staff who are required to work away from their home station for a 24 hour period are eligible to receive a taxable per diem in addition to their standard salary. Standard per diem rate for Solomon Islands Government employees is SBD $100.00 per day (Solomon Islands Government General Orders 2010). This SBD $100.00 rate has remained unchanged since 2006. Given historical SBD to USD exchange rates, the following unit costs have been assigned:   \| **Year** \| **SBD** \| **USD** \| \| --- \| --- \| --- \| \| 2008 \| $100.00 \| $13.70 \| \| 2009 \| $100.00 \| $13.14 \| \| 2010 \| $100.00 \| $12.75 \| \| 2011 \| $100.00 \| $13.32 \| \| 2012 \| $100.00 \| $14.11 \| |
| Equipment | Standard equipment required for the SDSS development in Solomon Islands includes additional laptop for staff based in provincial regions, programmed with basic software including the MS Office suite and MapInfo®. Generally each ‘region’ was given 2 PDAs enabled with GPS capacity which were required for the program staff to conduct geographical reconnaissance, and undertake brief household questionnaires. These PDAs were loaded with software including ArcGIS (and Arc Pad application) and MapInfo. Small portable generators were required for each GR team in order to power/charge laptops and PDAs. Extension cables, power boards and basic lighting equipment also required for GR teams whilst traveling in remote areas (to be used with portable generators). Additional printer (usually carried with GR team), and positioned in central location was also required for printing basic reports, household lists etc. Standard Equipment list based on experience in Solomon Islands. This equipment would be required for each ‘province’ with the number of ‘units’ dependent upon how many operational zones the GR is being conducted. Unit costs have been sourced from available data in AUD and SBD, and converted to 2008 USD for reference in the table below.   \| **Item** \| **Description** \| **Units** \| **AUD/SBD Value (year)** \| **USD 2008 Value** \| \| --- \| --- \| --- \| --- \| --- \| \| Laptop computer \| Durabook (Gamma-Tech Computer Corp., Fremont, CA) \| 1 \| AUD $2,140.00 (2008) \| $1,788.40 \| \| GIS integrated personal digital assistant (PDA) \| Trimble Juno ST (Trimble Navigation Limited, Sunnyvale, CA) \| 2 \| AUD $819.50 (2008) \| $1,369.72 \| \| Genset \| Yamaha, 2 Kva \| 1 \| SBD $9,000.00 (2010) \| $1,132.63 \| \| Peripherals \| Extension cable, power board (surge protected), portable light \| 1 \| SBD $344.00 (2010) \| $43.29 \| \| Desktop (portable) printer \|  \| 1 \| SBD $2,780.00 (2010) \| $360.90 \| \| **Total equipment package unit cost (2008 USD)** \| \| \| \| **$4,694.94** \| |
| Accommodation | Accommodation expenses incurred whenever inter- and intra- provincial travel is required. This varies significantly in Solomon Islands, depending on where teams and personnel are located. For example in a provincial capital, where there is access to transport, supplies and where most provincial administration occurs, per person per night accommodation can be up to SBD $500. However, when travelling to and within rural areas and remote villages – SBD $75.00 – SBD $100.00 are more often realistic per person per night costs.  For the purpose of this study accommodation costs for both Isabel and Temotu Province were collected over the period 2008 – 2011. These costs were collated and through consensus with program staff, and a midpoint or average per person, per night accommodation cost was assigned covering both provinces. A sample of cost information collected is detailed below, with the midpoint unit cost defined, represented in 2008 SBD.   \| TEMOTU PROVINCE \| ISABEL PROVINCE \| \| --- \| --- \| \| Rotary Lodge – 2010 – SBD $340.00 per night per person \| Mothers Union Rest House – 2010 – SBD $200 per night per person \| \| Luelta Resort – 2008 – SBD $160.00 \| Susubona Rest House – 2011 – SBD $100 p/n, p/p \| \| Anecdotal – 2008/2009 – SBD $75 - $100 p/n, p/p \| Tataba Rest House – 2011 – SBD $75 p/n, p/p \| \| **Midpoint: SBD $180.00** \| **Midpoint: SBD $180.00** \|   Once a midpoint or average was assigned, this 2008 cost figure was then converted to USD, and inflated/deflated using US CPI inflation data to provide a yearly accommodation unit cost, as per the table below:   \| **Year** \| **ACCOMMODATION MIDPOINT** \| \| \| --- \| --- \| --- \| \|  \| **SBD** \| **USD** \| \| 2008 \| $180.00 \| $24.65 \| \| 2009 \| $187.03 \| $24.57 \| \| 2010 \| $195.90 \| $24.97 \| \| 2011 \| $193.43 \| $25.76 \| \| 2012 \| $186.28 \| $26.29 \| |
| Other | This budget category was developed to cover expenses not routinely captured under the chart of accounts with the MHMS. These incidental costs were captured during the data collection process, and included in the overall cost summary for the SDSS development and implementation. |
| Boat/OBM hire | The Solomon Islands Government purchases boats and outboard motors (OBM) as the primary source of logistics support. Generally each division (within the Ministries) have their own boat(s) and OBM(s). In some instances, where these boats/OBMs are unavailable eg. where boats were already engaged in other activities, boats are hired by program staff from the community on a daily basis. These boat/OBM hire costs were captured during the data collection process, and included in the overall cost summary for the SDSS development and implementation. |
| Software | GIS compatible software is required for the operation of the SDSS. This software is considered an additional expenses outside of the project. Financial records were reviewed to obtain unit costs for each program utilised for the SDSS. These programs and their 2008 unit costs are detailed in the table below.   \| **Software Program** \| **2008 AUD unit cost** \| **2008 USD unit cost** \| \| --- \| --- \| --- \| \| MapInfo Professional 8.0 (Pitney Bowes Software Inc., Troy, NY). \| 50.14 \| $60.00 \| \| ArcPad 7.0 (ESRI, Redlands, CA) \| 27.08 \| $32.40 \| \| ArcGIS (ESRI, Redlands, CA) \| 62.5 \| $74.79 \| \| **Total Software Package Cost** \| $139.72 \| $167.19 \|   This cost was identified as a recurrent expenditure (annual licensing fees) to operate software on the various devices required for the SDSS (PDAs, laptops). The 2008 total software unit cost was used as a baseline, converted to US dollars and inflated each year based on US CPI inflationary figures.   \| **Year** \| **USD unit cost** \| \| --- \| --- \| \| 2008 \| $167.19 \| \| 2009 \| $166.60 \| \| 2010 \| $169.33 \| \| 2011 \| $174.67 \| \| 2012 \| $178.29 \| |
| Travel fares | Air fares are required in Solomon Islands to travel to provincial sites from Honiara. Costs for flights have been sourced from financial records of the NVBDCP and vary according to local and international fuel prices. These flight costs were also variable within the same year, based on the Solomon Airlines scheduling and availability.  Flight costs for Honiara to Buala (Isabel Province) and Honiara to Lata (Temotu Province) have been sourced from records obtained in 2010 (Temotu Province, 2010, SBD $3,787.00 and Isabel Province, 2010, SBD $1,671.80), used as the reference point and converted to 2010 USD, then inflated/deflated each year to provide unit costs for each year for travel fares. The following table has been used to reference unit costs for flights to Isabel and Temotu   \| **Year** \| **Isabel Province** \| \| **Temotu Province** \| \| \| --- \| --- \| --- \| --- \| --- \| \|  \| SBD \| USD (unit cost) \| SBD \| USD (unit cost) \| \| 2008 \| $1,532.42 \| $209.89 \| $3,569.15 \| $488.85 \| \| 2009 \| $1,592.24 \| $209.14 \| $3,615.43 \| $474.89 \| \| 2010 \| $1,667.80 \| $212.57 \| $3,787.00 \| $482.68 \| \| 2011 \| $1,646.75 \| $219.28 \| $3,739.20 \| $497.92 \| \| 2012 \| $1,585.86 \| $223.82 \| $3,600.94 \| $508.22 \| |
